# Impact of ultrasonographic blind spots for early-stage hepatocellular carcinoma during surveillance

**DOI:** 10.1371/journal.pone.0274747

**Published:** 2022-09-16

**Authors:** Junghwan Lee, Su Bee Park, Soyoung Byun, Ha Il Kim

**Affiliations:** 1 Department of Gastroenterology, Asan Medical Center, Seoul, South Korea; 2 Division of Gastroenterology, Department of Internal Medicine, Kyung Hee University Hospital at Gangdong, Seoul, South Korea; Yonsei University College of Medicine, REPUBLIC OF KOREA

## Abstract

**Background:**

Abdominal ultrasonography (US) is the backbone of hepatocellular carcinoma (HCC) surveillance. Although previous studies have evaluated clinical factors related to surveillance failure, none have focused specifically on US blind spots.

**Methods:**

This study included 1,289 patients who underwent 6 months intervals surveillance using US and serum alpha-fetoprotein (AFP) and were eventually diagnosed with single-nodular HCC. Patients were divided into US-detected group (n = 1,062) and US-missed group (HCC detected only by AFP ≥ 20ng/mL; n = 227). Blind spots consisted of four locations: hepatic dome, caudate lobe or around the inferior vena cava, <1 cm beneath the ribs, and the surface of the left lateral segment. Both groups were compared by HCC location, proportional distribution, treatment method, and overall survival.

**Results:**

A higher proportion of HCCs were located within blind spots in the US-missed group than in the US-detected group (64.3% vs. 44.6%, *P* < 0.001). HCC ≥ 2 cm detected in blind spots was higher than in non-blind areas (60.3% vs. 47.1%, *P =* 0.001). Blind spot HCCs were more treated with surgery, whereas those located in a non-blind area were more treated with local ablation. Patients with an HCC located within a blind spot in the US-detected group had better overall survival than the same in the US-missed group (*P* = 0.008).

**Conclusions:**

Using the current surveillance test, blind spots affected the initially detected HCC tumor size, applicability of the treatment modality, and overall survival. Physicians should pay attention to US blind spots when performing US-based HCC surveillance.

## Introduction

Hepatocellular carcinoma (HCC) is one of the most common cancers worldwide, and its early detection is crucial for patient survival [1, 2]. Consequently, global guidelines recommend surveillance every 6 months using abdominal ultrasonography (US)-based tests with or without serum alpha-fetoprotein (AFP) testing in high-risk populations [3–5]. Unfortunately, US-based HCC surveillance has been controversial for decades because of its suboptimal accuracy and actual contribution to prognosis [6–8].

US has an inherent limitation called “blind spots”, which are defined as the areas that could not be penetrated by acoustic waves or have a seriously hindered sonographic window because of their anatomic location [9–11]. Although numerous studies have evaluated clinical factors related to surveillance failure, none have specifically focused on US blind spots *per se* [7, 12–14]. Furthermore, no study has attempted to evaluate the effect of US blind spots on the prognosis of HCC detected under surveillance.

Another issue regarding surveillance tests is the applicability of curative treatment and its impact on survival [15]. In real-world settings, curative treatment methods are not equally applied to patients with HCC because tumor location and underlying liver function vary [16–18]. Accordingly, it is important to identify the effects of blind spots in terms of treatment selection and survival, especially in patients with early-stage HCC.

Here we examined the relationship between blind spots and clinical outcomes by evaluating cases of single-nodular HCC detected during regular surveillance. Using the current surveillance strategy, this study aimed to: 1) establish the effect of a blind spot location on tumor size and relevant clinical characteristics; and 2) examine whether blind spots affect treatment strategy and survival.

## Materials and methods

### Data collection and inclusion criteria

A total of 2,649 patients aged ≥ 20 years with HCC diagnosed at Asan Medical Center between January 2007 and December 2015 were included from the hospital-based HCC registry using the following exclusion criteria: (1) no history of undergoing surveillance for > 18 months prior to diagnosis; (2) HCC detected by cancer-related symptoms (weight loss, fatigue, malaise, pain, and loss of appetite); (3) absence of regular surveillance tests (≤ 6-month interval); (4) concurrent non-HCC malignancies; (5) prior abnormal surveillance results not evaluated by a diagnostic test, and (6) HCC detected unrelated to surveillance. The HCC diagnosis was based on pathological or radiological findings in accordance with international guidelines [4, 5]. All patients were categorized as high-risk (chronic hepatitis B infection, chronic hepatitis C infection, and liver cirrhosis), as defined by Korean practice guidelines [19]. All scans for diagnosis were performed within 1 month of the surveillance test (median [IQR], 2.0 [1.8–2.3] weeks).

**Fig 1:**
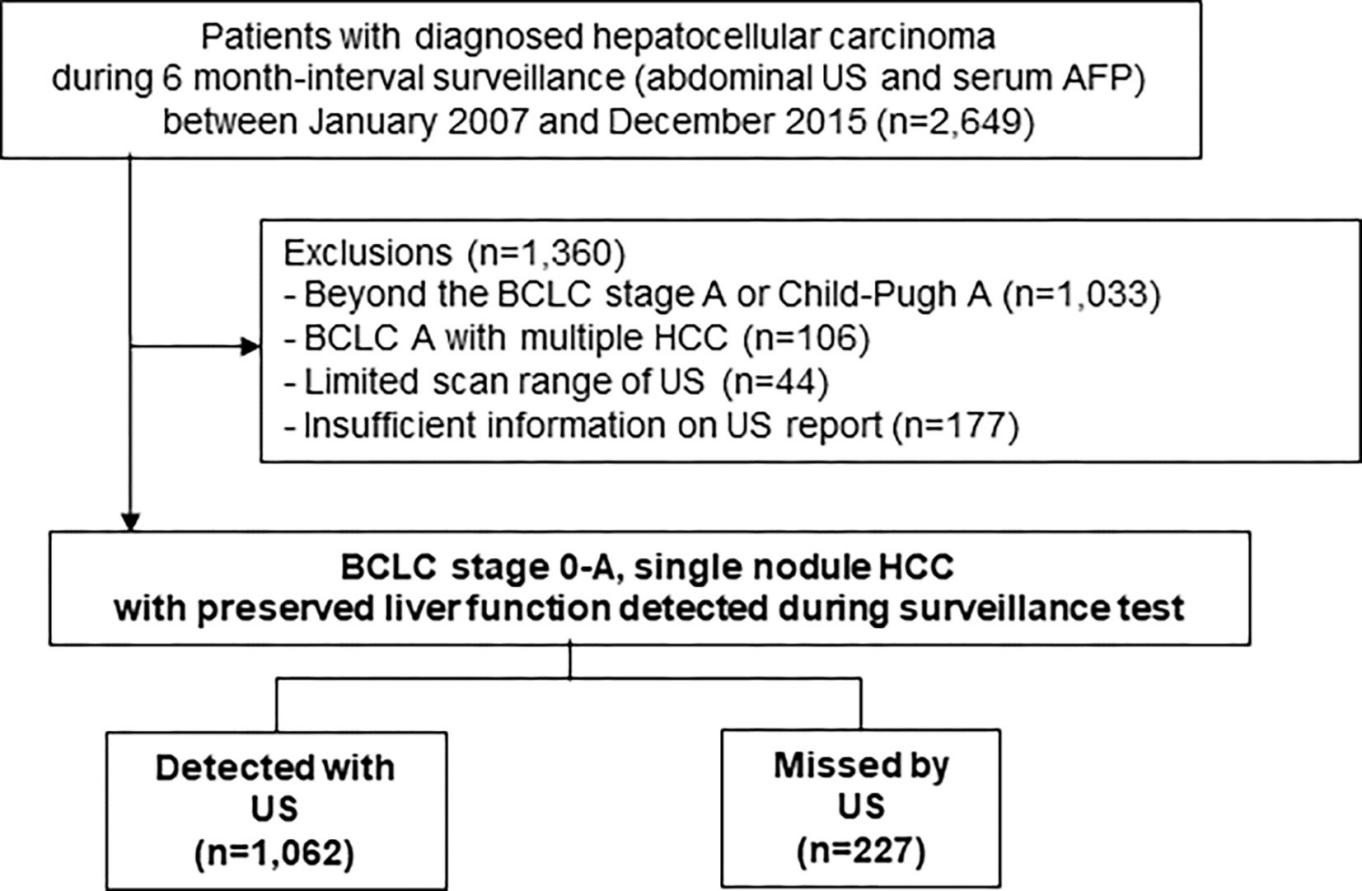
Study population flow sheet. AFP, serum alpha-fetoprotein; BCLC, Barcelona Clinic Liver Cancer; HCC, hepatocellular carcinoma; US, abdominal ultrasonography.

To evaluate the impact of US blind spots on the prognosis of early-stage HCC detected during surveillance, 1,360 patients were excluded as follows **(Fig 1)**: (1) 1,033 were diagnosed with HCC grades higher than Barcelona Clinic Liver Cancer (BCLC) stage A and Child-Pugh A, as they are not optimal candidates for curative treatment; (2) 106 patients with both BCLC stage A and multinodular HCC to ensure 1:1 tumor location matching between US and diagnostic images (i.e. computed tomography [CT] and/or magnetic resonance imaging [MRI]); and (3) patients with any scanning limitations (n = 44) as stated by the doctor who performed the US (poor patient cooperation, multinodular lesions on a cirrhotic background, and poor echogenic window). Moreover, we manually reviewed the US scanning images of the enrolled patients (by two researchers, JL and HIK, with over 5 years of US examination experience); consequently, 177 more patients were excluded because of an insufficient US scan, which was defined as the absence of one of the following items: 1) left subcostal scanning, 2) left longitudinal scanning, 3) epigastric transverse scanning, 4) epigastric longitudinal scanning, 5) right subcostal scanning (range of scanning between the bottom and right margin of the liver), 6) scanning of the hepatic dome (cranial part of the right and left hepatic lobes), 7) right intercostal scanning (including the right portal vein), and 8) right intercostal scanning (including the right hepatic vein) [11, 20]. Finally, we included patients who fulfilled the US scan range and were likely to undergo ultrasonography-based HCC surveillance without using additional diagnostic imaging such as dynamic CT or MRI [21–24]. This study was approved by the Institutional Review Board (no. 2020–0873) of the Asan Medical Center, and this study was conducted in accordance with the Declaration of Helsinki. Because this study is based on the retrospective analysis of existing clinical data, the requirement of obtaining informed patient consent was waived by the Institutional Review Board. This work was supported by grant from Medical Science Research Institute, Kyung Hee University Hospital at Gangdong in 2021.

### Clinical assessment

The included patients were classified into two groups according to the surveillance results prior to HCC confirmation as follows: 1) US-detected group (n = 1,062), which included patients with suspected malignant lesions detected by US with or without AFP increase, and 2) US-missed group (n = 227), which included patients with a high serum AFP test score (≥20 ng/mL) and no focal lesions on US [25, 26]. Detailed information including demographics, clinical data, tumor characteristics, and survival outcomes were extracted from inpatient and outpatient medical records using the anonymized clinical database system of our institution (ABLE) and the database of the National Population Registry of the Korea National Statistical Office using the patients’ unique personal identification numbers [27].

### Study definition of US findings

We adopted the classical definition of blind spots as follows: 1) hepatic dome (right and left), 2) caudate lobe or around the inferior vena cava (IVC), 3) <1 cm beneath either right or left ribs, and 4) surface of the left lateral segment (segments 2 and 3) [9, 11]. The HCC locations were confirmed by review of the electronic US and diagnostic images (dynamic contrast-enhanced CT or MRI). Subsequently, the location of each HCC lesion was reviewed manually. If the HCC mass was included in the blind spot, it was classified as being located in the blind spot, regardless of size or tumor morphology. Whenever an HCC was located in both blind spots 1 and 3 simultaneously, it was categorized as blind spot 1 (hepatic dome). Similarly, if the HCC was in blind spot 3 and blind spot 4 simultaneously, it would be classified as blind spot 4 (surface of the left lateral segment). Liver cirrhosis was defined as the presence of one of the following items found in a cirrhosis background: coarse parenchymal texture, surface nodularity, blunting edge, and splenomegaly [28, 29]. Signs of fatty liver included a bright parenchyma, a high liver-to-kidney contrast, deep beam attenuation, and blurred vessel walls [28].

### Statistical analysis

Patient characteristics at baseline were compared using the chi-squared test or Fisher’s exact test to examine relationships between categorical variables, and Student’s *t-test* or Wilcoxon rank-sum test to compare mean values of continuous variables. Clinical variables associated with blind spots were analyzed using logistic regression analysis. A Kaplan-Meier analysis was used to compare the overall survival between the US-detected and US-missed groups, which is defined as the interval between the date of HCC diagnosis and death of any cause. Death, survival, and follow-up data were fully accessible through the registry of the Asan Medical Center and collected until December 31, 2019. A Cox proportional hazard model with backward elimination was used to identify the independent characteristics of the blind spots associated with overall survival. Potential confounders (*P* < 0.10) among the variables in the univariate model were used as input variables in the multivariate analysis. All statistical analyses were performed using R statistical software (version 4.0.2; R Foundation Inc.; http://cran.r-project.org). The threshold for statistical significance was set at *P* < 0.05.

## Results

### Patient characteristics and tumor distribution

In this study, 1,289 patients with single-nodular BCLC stage 0-A HCC detected during surveillance were enrolled. All study patients had a Child-Pugh class A classification as described above. Most patients (n = 964 [74.8%]) were men. Hepatitis B virus infection was the predominant etiology of liver disease (n = 1,067 [82.8%]). The mean age was higher in the US-detected group than in the US-missed group (57.0 vs. 55.2). The proportion of men was higher in the US-detected group than in the US-missed group (77.9% vs. 60.4%, *P* < 0.001). There were no statistically significant differences in diabetes mellitus, hypertension, alcohol consumption, or liver disease etiology between the US-detected (n = 1,062 [82.4%]) and US-missed (n = 227 [17.6%]) groups. Mean body mass index, international normalized ratio, serum creatine, albumin, aspartate aminotransferase, and alanine aminotransferase values did not significantly differ between the groups. The median AFP value did not significantly differ between the groups. The mean platelet count, representing underlying portal hypertension, was significantly lower in the US-missed group than in the US-detected group (*P* < 0.001). A higher proportion of patients in the US-missed versus US-detected group underwent surveillance at a tertiary referral hospital (36.5% vs. 48.9%, *P* = 0.001). Cirrhotic features were more commonly noted on US in the US-missed group than in the US-detected group (68.7% vs. 56.9%, *P* = 0.013), while fatty changes were seen more often in the US-detected group than in the US-missed group (11.6% vs. 4.0%, *P* = 0.001) **(Table 1)**.

**Table 1:** Characteristics of 1,289 enrolled patients with Barcelona Clinic Liver Cancer stage 0-A single-nodular hepatocellular carcinoma detected during US surveillance.

	US-detected group (n = 1,062)	US-missed group (n = 227)	*P* value	Total (N = 1,289)
Age (years)	57.0 ± 9.4	55.2 ± 10.5	0.012	56.7 ± 9.6
Male	827 (77.9%)	137 (60.4%)	<0.001	964 (74.8%)
Body mass index (kg/m^2^)	24.6 ± 3.0	24.8 ± 3.0	0.296	24.6 ± 3.0
Diabetes mellitus	195 (18.4%)	34 (15.0%)	0.226	229 (17.8%)
Hypertension	291 (27.4%)	71 (31.3%)	0.238	362 (28.1%)
Alcohol consumption	450 (42.4%)	83 (36.6%)	0.107	533 (41.3%)
HBV infection	878 (82.7%)	189 (83.3%)	0.832	1067 (82.8%)
HCV infection	84 (7.9%)	26 (11.5%)	0.083	110 (8.5%)
Platelet count (×10^3^/mm^3^)	140.8 ± 56.0	124.4 ± 51.3	<0.001	137.9 ± 55.5
International normalized ratio	1.07 ± 0.09	1.08 ± 0.09	0.127	1.07 ± 0.09
Serum creatinine (mg/dL)	0.9 ± 0.5	0.9 ± 0.6	0.746	0.9 ± 0.5
Serum albumin (g/dL)	3.9 ± 0.4	3.9 ± 0.4	0.522	3.9 ± 0.4
Serum bilirubin (mg/dL)	0.9 ± 0.4	0.9 ± 0.4	0.819	0.9 ± 0.4
Serum AST (IU/L)	52 ± 39	52 ± 36	0.943	52 ± 39
Serum ALT (IU/L)	43 ± 33	47 ± 40	0.084	44 ± 35
AFP (ng/mL)*	8.1 (3.4–35.2)	164.1 (68.4–451.1)	<0.001	12.4 (4.1–92.0)
AFP (log_10_ng/mL)*	0.91 (0.53–1.55)	2.21 (1.84–2.65)	<0.001	1.09 (0.61–1.96)
Surveillance at tertiary referral hospital	388 (36.5%)	111 (48.9%)	0.001	499 (38.7%)
Liver cirrhosis on US	636 (59.9%)	156 (68.7%)	0.013	792 (61.4%)
Fatty liver on US	123 (11.6%)	9 (4.0%)	0.001	132 (10.2%)

AFP, alpha-fetoprotein; BCLC, Barcelona Clinic Liver Cancer; ALT, alanine transaminase; AST, aspartate transaminase; HBV, hepatitis B virus; HCC, hepatocellular carcinoma; HCV, hepatitis C virus; US, ultrasonography

*Median (interquartile range)

**Table 2** shows the comparison of the tumor characteristics between the US-detected group and the US-missed group. Although the mean HCC size was significantly higher in the US-detected group than that in the US-missed group (2.5 ± 1.7 vs. 2.2 ± 1.2 cm, P < 0.001), there was no difference in median size between the two groups (2.0 cm). The proportion of infiltrative HCC lesions did not differ between the groups (0.8% vs. 0.9%, P = 0.603); however, the size of infiltrative HCC in the US-detected group was higher than that in the US-missed group (**Table 2**). Focusing on the US-blind spots, the mean tumor size was increased in both the US-detected group and the US-missed group; however, the mean and the median tumor sizes in the US-detected group was larger than that in the US-missed group [mean tumor size, 2.6 vs. 2.3 cm, P *=* 0.004; median (range) tumor size, 2.2 (1.0–11.5) vs. 2.0 (1.0–6.5)]; among them, 2 out of 4 (in the hepatic dome and caudate lobe or around the IVC) were significantly larger in the US-detected group than in the US-missed group (2.7 ± 1.4 vs. 2.1 ± 1.1 cm, *P* = 0.009; 3.4 ± 1.9 vs. 2.4 ± 1.0 cm, *P* = 0.017).

**Table 2:** Distribution of hepatocellular carcinoma tumors by characteristic and blind spot location on ultrasonography.

	US-detected group	US-missed group	*P* value
All HCC tumors	n = 1,062	n = 227	
Size (cm)	2.5 (1.7) 2.0 (1.5–3.0)*	2.2 (1.2) 2.0 (1.3–2.8)*	<0.001†
Infiltrative type‡	9 (0.8%)	2 (0.9%)	0.603
**HCC tumors within blind spots**	474 (44.6%)	146 (64.3%)	<0.001
Size (cm)	2.6 (1.5) 2.2 (1.6–3.3)*	2.3 (1.2) 2.0 (1.4–3.0)*	0.004†
Infiltrative type‡	6 (1.3%)	2 (1.4%)	0.597
Each blind spot			
(1) hepatic dome	201 (18.9%)	50 (22.0%)	
size (cm)	2.7 ± 1.4	2.1 ±1.1	0.009
(2) caudate lobe or around IVC	28 (2.6%)	25 (11.0%)	
size (cm)	3.4 ± 1.9	2.4 ± 1.0	0.017
(3) <1 cm beneath ribs	219 (20.6%)	56 (24.7%)	
size (cm)	2.4 ± 1.5	2.3 ± 1.2	0.490
(4) left lateral segment, surface	26 (2.4%)	15 (6.6%)	
size (cm)	3.1 ± 1.3	2.7 ± 1.8	0.462

Data are presented as number (%) or mean (standard deviation, SD), unless otherwise indicated.

*Median (interquartile range)

† Student’s t-test between mean tumor sizes.

‡The total number of infiltrative HCCs was 11 (size [cm]; mean[SD],8.1 [3.1]; median[IQR],7.0[5.5–11.5]). Nine were in the US-detected group (mean[SD]:8.7[3.0]; median[IQR]:7.2 [6.1–11.8], and 2 were in the US-missed group (4.5 cm and 5.7 cm)

HCC, hepatocellular carcinoma; IVC, inferior vena cava; US, ultrasonography

The proportion of tumors in blind spots was significantly higher in the US-missed group than that in the US-detected group (64.3% vs. 44.6%, *P* < 0.001, **Table 2**). When the analysis limited HCC to < 2 cm on blind spots, the results showed a similar distribution between the US-missed group and US-detected group (59.8% vs. 37.1%, *P* < 0.001), and the proportion of US-missed group of blind spots 2 and 4 was higher than that of tumors of all sizes (**S1 Table in S1 File**). The results of logistic regression analyses showed that all blind spots were independently associated with the US-missed group; in particular, blind spots 2 and 4 [odds ratio (95% confidence interval), 1.903 (1.251–2.895) for blind spot 1; 7.875 (4.029–15.391) for blind spot 2; 1.643 (1.094–2.467) for blind spot 3; and 3.516 (1.655–7.471) for blind spot 4; all *P* < 0.05] (**S2 Table in S1 File**).

### Impact of blind spot location on HCC size and relevant clinical factors

**Fig 2:**
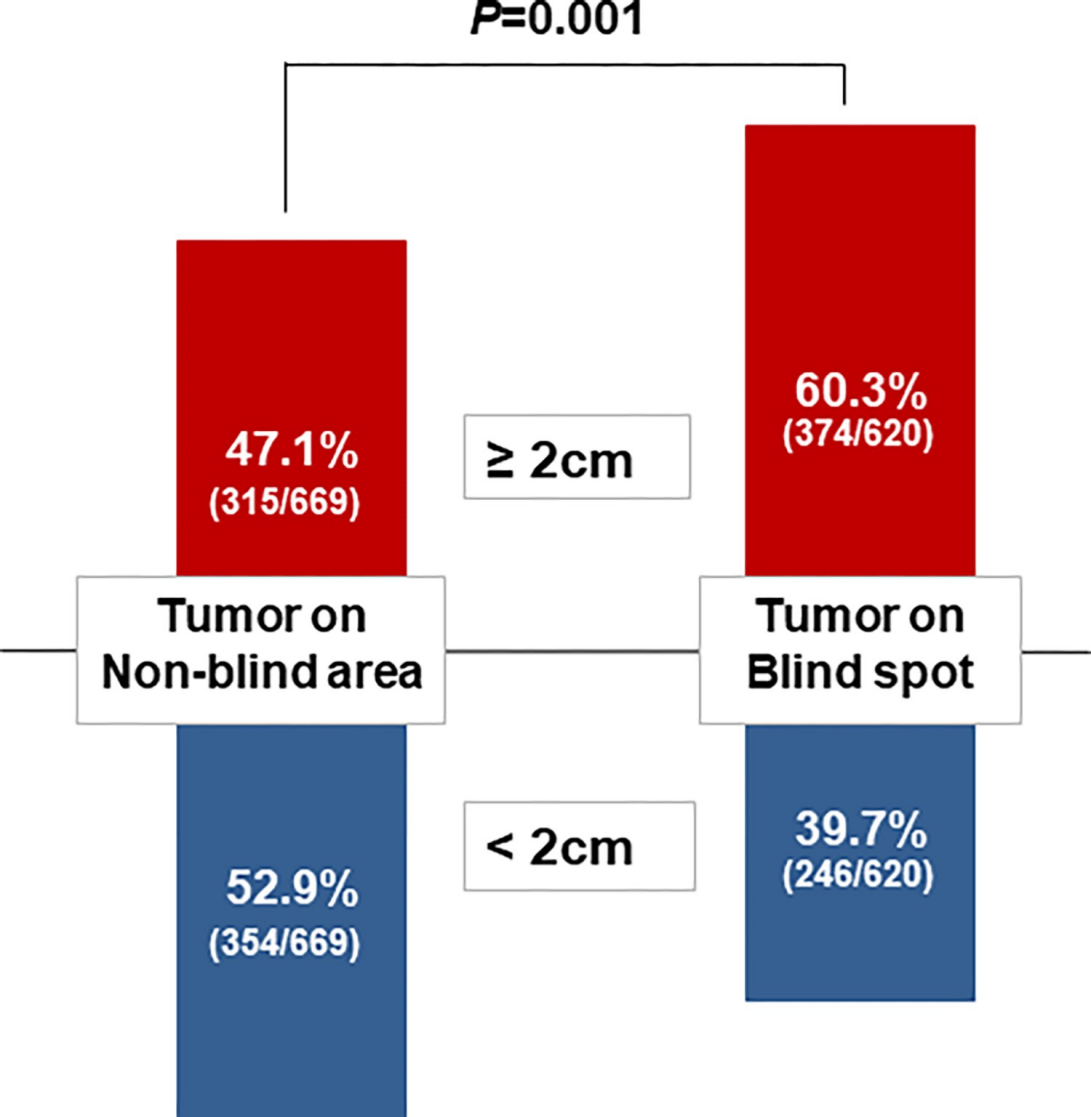
Comparison of the size distribution of HCC by blind vs. non-blind spot location. HCC, hepatocellular carcinoma.

**Fig 3:**
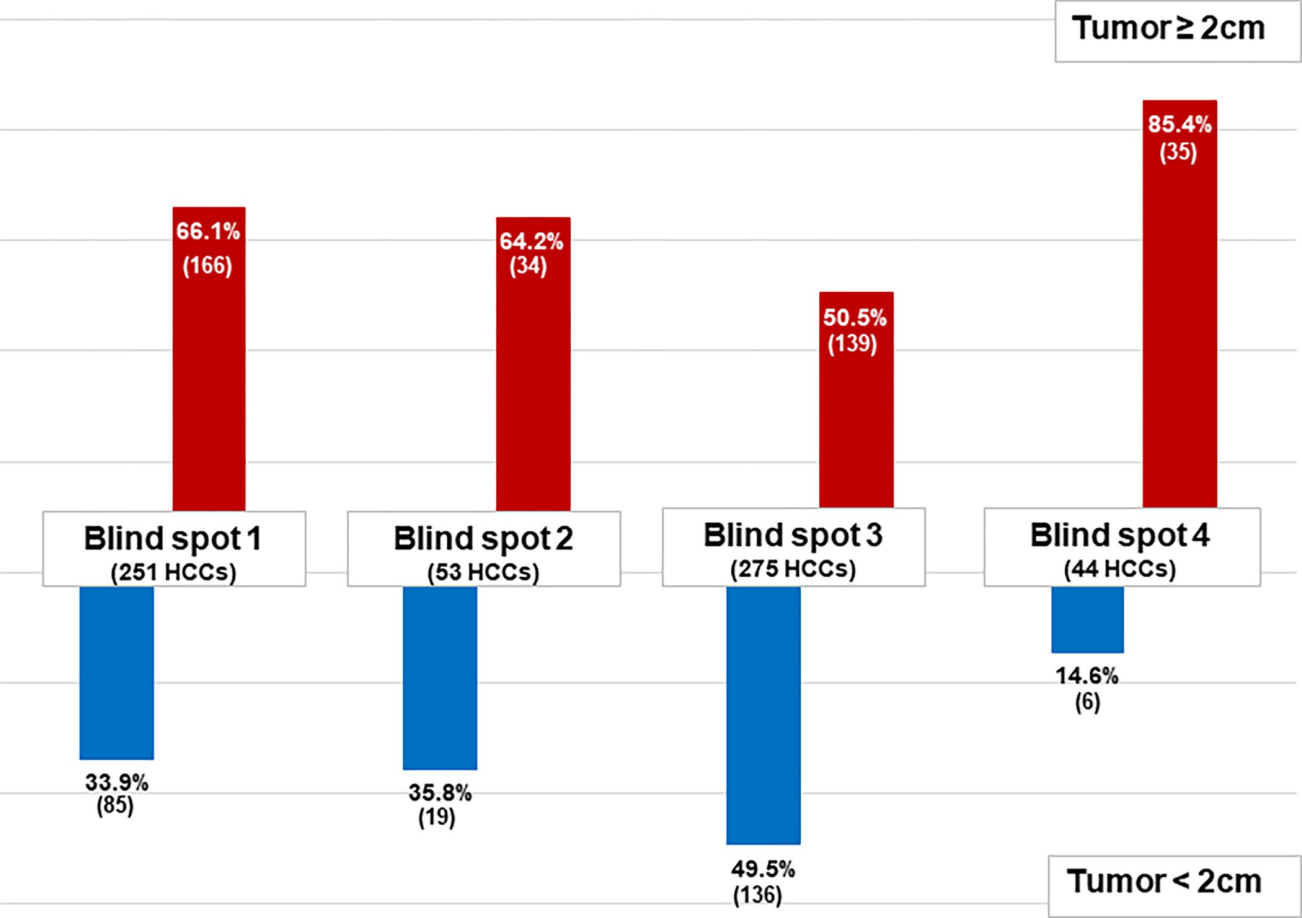
Comparison of the size distribution of HCC in blind spots of the total study population. HCC, hepatocellular carcinoma; US, ultrasonography; *Definition of blind spots: hepatic dome (blind spot 1), caudate lobe or around the inferior vena cava (blind spot 2), <1 cm beneath the ribs (blind spot 3), and surface of the left lateral segment (blind spot 4).

Overall, a significantly higher proportion of HCC tumors ≥ 2 cm were detected within blind spots than in non-blind spots (60.3% vs. 47.1%, *P* = 0.001) **(Fig 2)**. When the blind spot locations were separated into four groups **(Fig 3)**, the proportion of HCC tumors ≥ 2 cm was higher for all four, but prominent in blind spot 4 (66.1% vs. 33.9% for blind spot 1, 64.2% vs. 35.8% for blind spot 2, 50.5% vs. 49.5% for blind spot 3, and 85.4% vs. 14.6% for blind spot 4). These trends were maintained in the US-missed group **(S1A Fig in S1 File)** and the US-detected group **(S1B Fig in S1 File)**, and the US-detected group had a higher proportion of HCC tumors ≥ 2 cm than the US-missed group for all four blind spots.

**Table 3** shows the relationship between patient characteristics and HCC tumors located within blind spots. In univariate analysis, male sex, alcohol consumption, AFP > 200 ng/mL, HCC ≥ 2 cm, cirrhotic features on US, and HCC detected on US were significantly associated with an HCC location within a blind spot. After the adjustment for confounders, male sex (odds ratio [OR], 0.764; 95% confidence interval [CI], 0.589–0.992; *P* = 0.043), HCC size ≥ 2 cm (OR, 1.790; 95% CI, 1.428–2.245; *P* < 0.001), cirrhotic features (OR, 1.297; 95% CI, 1.029–1.636; *P* = 0.028), and HCC missed by US (OR, 2.177; 95% CI, 1.581–2.998; *P* < 0.001) were independent factors associated with a blind spot tumor location.

**Table 3:** Baseline parameters related to detection of hepatocellular carcinoma tumors located within blind spots during surveillance.

Variable	Unadjusted		Adjusted	
	OR (95% CI)	*P* value	OR (95% CI)	*P* value
Age ≥ 60 years	0.892 (0.710–1.119)	0.322		
Male sex	0.699 (0.543–0.900)	0.005	0.764 (0.589–0.992)	0.043
BMI ≥ 30 kg/m^2^	1.224 (0.698–2.144)	0.481		
HBV infection	0.995 (0.745–1.329)	0.974		
HCV infection	1.131 (0.765–1.672)	0.538		
Alcohol consumption	0.790 (0.632–0.987)	0.038	0.909 (0.711–1.161)	0.444
Platelet count < 100k/mm^3^	1.089 (0.846–1.401)	0.510		
Serum AST > 40 IU/L	1.029 (0.827–1.281)	0.797		
Serum ALT > 40 IU/L	0.946 (0.756–1.182)	0.623		
AFP > 200 ng/mL	1.289 (0.966–1.720)	0.084	0.945 (0.689–1.295)	0.724
Surveillance performed at tertiary referral hospital	1.082 (0.864–1.353)	0.493		
HCC tumor size ≥ 2 cm	1.709 (1.369–2.132)	<0.001	1.790 (1.428–2.245)	<0.001
Cirrhosis on US	1.285 (1.026–1.609)	0.029	1.297 (1.029–1.636)	0.028
Fatty liver on US	0.888 (0.619–1.275)	0.521		
HCC missed by US	2.236 (1.661–3.010)	<0.001	2.177 (1.581–2.998)	<0.001

ALT, alanine transaminase; AFP, alpha-fetoprotein; AST, aspartate transaminase; BMI, body mass index; CI, confidence interval; HBV, hepatitis B virus; HCC, hepatocellular carcinoma; HCV, hepatitis C virus; OR, odds ratio; US, ultrasonography

### Impact of blind spot tumor location on initial treatment of HCC by tumor size

Of the total 1,289 patients, 1,008 (78.2%) underwent initial curative treatment (hepatectomy, radiofrequency ablation [RFA], or liver transplantation) during the study period **(Table 4)**. The proportion of patients who received curative treatment did not significantly differ between blind spot and non-blind location regardless of HCC size (78.1% vs. 78.3% for any size, *P* = 0.910; 76.4% vs. 77.7% for HCCs < 2 cm, *P* = 0.717).

**Table 4:** Differences in initial treatment by HCC tumor size and location.

**All HCC tumors**						
	Any size			<2 cm		
	Within a blind spot (n = 620)	In a non-blind area (n = 669)	*P* value	Within a blind spot (n = 354)	In a non-blind area (n = 246)	*P* value
Curative treatment	484 (78.1%)	524 (78.3%)	0.910	188 (76.4%)	275 (77.7%)	0.717
Hepatectomy	372 (60.0%)	308 (46.0%)	<0.001	113 (45.9%)	105 (29.7%)	<0.001
RFA	105 (16.9%)	204 (30.5%)	<0.001	72 (29.3%)	162 (45.8%)	<0.001
LT	6 (1.0%)	9 (1.3%)	0.528	2 (0.8%)	6 (1.7%)	0.293
**US-detected group**						
	Any size			<2 cm		
	Within a blind spot (n = 474)	In a non-blind area (n = 588)	*P* value	Within a blind spot (n = 182)	In a non-blind area (n = 311)	*P* value
Curative treatment	377 (79.5%)	459 (78.1%)	0.559	142 (78.0%)	238 (76.5%)	0.703
Hepatectomy	288 (60.8%)	268 (45.6%)	<0.001	83 (45.6%)	89 (28.6%)	<0.001
RFA	86 (18.1%)	180 (30.6%)	<0.001	58 (31.9%)	142 (45.7%)	0.003
**US-missed group**						
	Any size			<2 cm		
	Within a blind spot (n = 146)	In a non-blind area (n = 81)	*P* value	Within a blind spot (n = 64)	In a non-blind area (n = 43)	*P* value
Curative treatment	107 (73.3%)	65 (80.2%)	0.241	46 (71.9%)	37 (86.0%)	0.085
Hepatectomy	84 (57.5%)	40 (49.4%)	0.237	30 (46.9%)	16 (37.2%)	0.322
RFA	19 (13.0%)	24 (29.6%)	0.002	14 (21.9%)	20 (46.5%)	0.007

HCC, hepatocellular carcinoma; LT, liver transplantation; RFA, radiofrequency ablation; US, ultrasonography

A significantly higher proportion of patients with tumors located within blind spots underwent hepatectomy than those with tumors in non-blind areas; in contrast, patients with HCC tumors located in non-blind areas were more frequently treated with RFA. These trends were maintained in patients with HCC detected by US. In the US-missed group, RFA was more frequently performed for HCC tumors located in non-blind areas than in blind spots regardless of size (29.6% vs. 13.0% for any size, *P =* 0.002; 46.5% vs. 21.9% for HCC < 2 cm, *P =* 0.007). However, the proportions of patients requiring hepatectomy did not differ significantly between those with tumors located in blind spots and those with tumors in non-blind areas regardless of size (*P* = 0.237 for any HCC size; *P* = 0.322 for HCC tumors < 2 cm).

### Association between blind spot tumor location and survival outcomes

**Fig 4:**
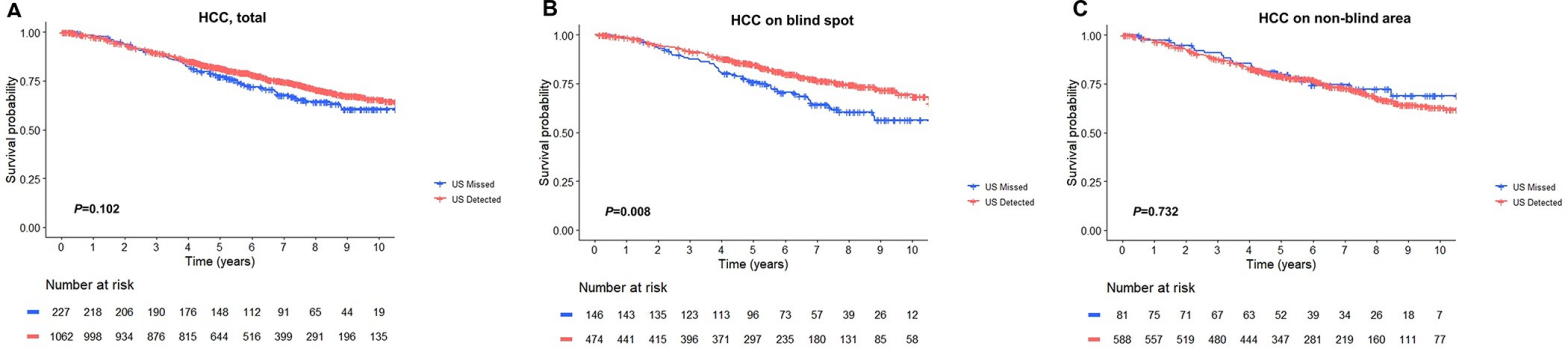
Overall survival of the US-detected versus US-missed groups: (A) entire study population, (B) HCC in a blind spot, and (C) HCC in a non-blind area (*P* value calculated by the log-rank test).

During a median follow-up of 5.91 years (interquartile range, 4.11–8.22 years), 336 patients (26.1%) died of any cause; of them, 264 (78.6%) were in the US-detected group and 72 (21.4%) were in the US-missed group. The 5-year cumulative overall survival rates estimated by the Kaplan-Meier method were 81.6% and 76.6% for the US-detected and US-missed groups, respectively. There was no significant intergroup difference in overall survival (log-rank test, *P* = 0.102) **(Fig 4A)**. When an HCC tumor was located in a blind spot, the overall survival rate was significantly lower in the US-missed group than in the US-detected group (*P* = 0.008) **(Fig 4B)**. In contrast, there was no significant intergroup difference in overall survival when an HCC tumor was detected in a non-blind area (*P* = 0.732) **(Fig 4C)**. Univariate and adjusted analyses were performed to identify significant variables related to the time-dependent outcomes **(Table 5)**. After adjusting for confounders, age ≥ 60 years (hazard ratio [HR], 1.454; 95% CI, 1.154–1.832; *P* = 0.002), hepatitis B virus infection (HR, 0.542; 95% CI, 0.416–0.705; *P* < 0.001), platelet count < 100 k/mm^3^ (HR, 1.452; 95% CI, 1.149–1.834; *P* = 0.002), AFP > 200 ng/mL (HR, 1.430; 95% CI, 1.095–1.867; *P* = 0.009), HCC size ≥ 2 cm (HR, 1.420; 95% CI, 1.141–1.766; *P* = 0.002), and initial curative treatment (HR, 0.448; 95% CI, 0.357–0.564; *P* < 0.001) were independently associated with survival in patients with single-nodular HCC detected during surveillance. Analysis of only HCCs located in blind spots, age ≥ 60 years (HR, 1.916; 95% CI, 1.365–2.689; *P* < 0.001), hepatitis C virus infection (HR, 1.747; 95% CI, 1.096–2.786; *P* = 0.019), HCC-missed group (HR, 1.413; 95% CI, 1.003–1.990; *P* = 0.048), and initial curative treatment (HR, 0.404; 95% CI, 0.290–0.563; *P*< 0.001) were independently associated with survival (**S3 Table in S1 File**).

**Table 5:** Factors associated with overall survival in patients with single-nodular HCC detected during US surveillance.

Variable	Unadjusted		Adjusted	
	HR 95% CI	*P*	HR (95% CI)	*P*
Age ≥ 60 years	1.720 (1.388–2.133)	<0.001	1.454 (1.154–1.832)	0.002
Male sex	1.117 (0.867–1.440)	0.390		
BMI ≥ 30 kg/m^2^	1.525 (0.936–2.486)	0.090	1.278 (0.779–2.095)	0.331
HBV infection	0.500 (0.391–0.638)	<0.001	0.542 (0.416–0.705)	<0.001
HCV infection	2.117 (1.563–2.868)	<0.001	1.308 (0.868–1.971)	0.199
Alcohol consumption	1.197 (0.965–1.485)	0.102		
Platelet count < 100^3^/mm^3^	1.615 (1.288–2.024)	<0.001	1.452 (1.149–1.834)	0.002
Serum AST > 40 IU/L	1.151 (0.930–1.426)	0.197		
Serum ALT > 40 IU/L	1.182 (0.953–1.467)	0.128		
AFP >200 ng/mL	1.351 (1.037–1.761)	0.026	1.430 (1.095–1.867)	0.009
Surveillance at tertiary referral hospital	1.017 (0.819–1.263)	0.878		
HCC tumor size ≥ 2 cm	1.337 (1.077–1.660)	0.009	1.420 (1.141–1.766)	0.002
Cirrhosis on US	1.143 (0.914–1.431)	0.241		
Fatty liver on US	1.065 (0.751–1.512)	0.724		
HCC detected by US	0.805 (0.620–1.045)	0.103		
Tumor within a blind spot	0.901 (0.727–1.117)	0.341		
Initial curative treatment	0.402 (0.322–0.502)	<0.001	0.448 (0.357–0.564)	<0.001

AFP, alpha-fetoprotein; ALT, alanine transaminase; AST, aspartate transaminase; BMI, body mass index; CI, confidence interval HBV, hepatitis B virus; HCC, hepatocellular carcinoma; HCV, hepatitis C virus; HR, hazard ratio; US, ultrasonography

## Discussion

Because of the complexity of factors affecting HCC surveillance outcomes in actual practice, no study has focused on the effects of blind spots [6, 7, 12–14]. In contrast to the benefit of surveillance tests for all HCC stages there have been limited data regarding the early-stage HCC during surveillance tests, and whether relevant factors are positively or negatively affected by surveillance remains unclear [8, 12, 30, 31]. Here we analyzed 1,289 consecutive patients diagnosed with single-nodular HCC during surveillance tests to determine whether blind spots could hinder the detection of early-stage HCC. When selecting the optimal US-based surveillance candidates, we focused on the following: 1) evaluating the role of the blind spot while excluding confounding variables, 2) excluding factors influencing treatment decisions, and 3) including the population that underwent US-based HCC surveillance without diagnostic imaging (i.e., CT or MRI) due to insufficient scan range by US. In our study, HCC tumors detected in the US-detected group, specifically those located in blind spots, were significantly larger in diameter than those in the US-missed group. Furthermore, a significantly higher proportion of HCC tumors > 2 cm were located in blind spots than in non-blind areas, limiting our analysis only to HCCs located in the four blind spots, the size of HCCs was consistently higher in both the US-detected and US-missed groups. These findings suggest that the blind spot location made it difficult to find very early HCC tumors on US, even when patients underwent properly applied US-based surveillance. Although the US-missed group, defined by AFP positivity (≥20 ng/mL) alone, compensated for US missing HCC, was considered insufficient and needed optimizing.

Various studies have evaluated relevant factors associated with surveillance failure, including patient characteristics, screening adherence, and US quality [6, 32–34]. However, no studies have focused on US blind spots. We evaluated well-known confounders of US-based surveillance to determine whether they were related to the detection of HCC on US-blind spots. In our study, women, HCC ≥2 cm, cirrhosis, and the US-missed group were significantly associated with HCC located in blind spots. Although tumor location in blind spots was not necessarily equal to surveillance failure: these results suggested that US-based tests could be hindered by these factors, even in optimal candidates for surveillance. Another problem is that no studies to date have analyzed the pooled effect of the four blind spots. The hepatic dome (blind spot 1) is reportedly associated with US-based surveillance failure; however, other well-known blind spots have never been studied [35–37]. Analysis of each blind spot revealed that the proportion of HCC tumors ≥ 2 cm was consistently higher in all blind spots. In particular, the proportion of HCC tumors ≥ 2 cm was highest on the surface of the left lateral segment (blind spot 4). This finding indicates that physicians should pay more attention to US blind spots, even in optimal candidates. Furthermore, the detection of HCC tumors in blind spots could be much more dependent on AFP tests during surveillance. Therefore, the use of AFP needs to be optimized, and future studies are needed to evaluate the efficacy of different tumor markers in HCC surveillance [38, 39].

HCC location can affect the treatment method [16–18]. There are currently three options for the curative treatment of HCC: surgical resection, local ablation (e.g., RFA), and liver transplantation [3–5]. In most cases, surgical resection and local ablation are realistic options for the curative treatment of HCC because liver transplantation is not always applicable in actual clinical situations due to organ unavailability [15]. In our study, the total curative treatment portion did not differ between the HCC tumors located in blind spots and those in non-blind areas. However, hepatectomy was more frequently performed for HCC tumors within blind spots; in contrast, RFA was more frequently performed for HCCs in non-blind areas. The preference for hepatectomy for HCC tumors within blind spots is attributed to the accessibility of the probe, which could make it difficult to perform RFA as reported in previous studies [17, 40, 41]. Interestingly, the aforementioned tendency remained regardless of tumor size in the US-detected and US-missed groups. Focusing on blind spots, adjusted logistic regression analysis indicated that HCC tumors detected within blind spots could be larger and have a cirrhotic background, which could complicate both RFA and hepatectomy.

The goal of surveillance tests is to improve prognosis by early detection and ultimately achieve survival benefits [2]. Curative treatment alone does not always warrant survival benefits; therefore, we evaluated whether the blind spot location affected overall survival in patients of the US-detected and US-missed groups. In the analysis of total HCC cases, there was no difference in overall survival between the US-detected and US-missed groups. When analyzing the patients with HCC tumors located within a blind spot, the US-missed group had a statistically significant decrease in survival rate when compared to the US-detected group. However, this trend was not observed in patients with HCCs located in non-blind areas. Multivariable regression analyses showed that overall survival was independently associated with tumor size and liver cirrhosis, but not with the blind spot itself. Interestingly, when the analysis for HCC tumors in blind spots was performed, the US-missed group was independently associated with overall survival. These results suggest that tumor size affects survival; however, once a tumor is detected, various clinical factors (such as age, thrombocytopenia, serum AFP level, and initial curative treatment) may also contribute to survival. According to our study results, the size of HCC detected in the blind spots was larger than that of HCC in non-blind areas, but the overall survival could not be explained by the size alone. Further studies are necessary to confirm this in advanced stage cases of HCC with minimized US-limitation.

This study had some limitations. First, this was a retrospective study, and US quality was evaluated based on the captured images only. In addition, due to the exclusion of suboptimal candidates for US-based surveillance, our results should be interpreted with caution. Second, it is unknown whether US providers performed the US when blind to the AFP results. Furthermore, we could not analyze the experience of the US provider and whether the test was carried out under the standard protocol. Instead, the US quality was evaluated based on the existence of a specific US image as the standard if there was no record of the US image, the subject was excluded from the study. Interestingly, whether patients underwent surveillance at a tertiary referral hospital was not associated with either tumor location or patient survival in the adjusted regression analyses. These results suggest that if suboptimal US candidates or non-standardized exams are avoided in regular surveillance, the quality issue may be minimized [11, 20]. However, a further study needs to validate the issue of generalizability in terms of etiology of liver disease, hepatic function, stage of HCC, and technical issue of ultrasonography. Third, HCC tumors tended to be less common in US-blind spots in male patients; it is necessary to confirm whether this trend would be replicated in the entire surveillance population. Fourth, we could not evaluate alternative methods to refine HCC detection in US-missed group. The cost-effectiveness of an alternative method for measuring tumor markers (e.g., longitudinal AFP measurement or concomitant use of multiple tumor markers) will require evaluation in future studies. Lastly, this study did not focus on the screening accuracy of US or AFP; rather, it analyzed single-nodular HCC cases from hospital-based registry. Moreover, this study included HCC cases that could be analyzed 1:1 comparison in terms of tumor location. Whether tumors being located within blind spots impacts surveillance effectiveness requires evaluation in further studies, especially in cases of advanced HCC.

## Conclusions

In the current study of HCC patients who underwent regular surveillance, US blind spots affected the initially detected HCC tumor size, treatment modality applicability, and overall survival. Our findings suggest that physicians should pay attention to US blind spots and strive to establish an individualized strategy for using tumor markers to detect early-stage HCC.

## Supporting information

S1 File (DOCX)

## List of abbreviations

ABLE: Anonymized clinical database system of our institution
ALT: alanine transaminase
AST: aspartate transaminase
AFP: serum alpha-fetoprotein
BCLC: Barcelona Clinic Liver Cancer
BMI: body mass index
CT: computed tomography
HBV: hepatitis B virus
HCV: hepatitis C virus
HCC: Hepatocellular carcinoma
HR: hazard ratio
IVC: inferior vena cava
LT: liver transplantation
MRI: magnetic resonance imaging
OR: odds ratio
RFA: radiofrequency ablation
US: abdominal ultrasonography
