# Supplementary material for: Impact of ultrasonographic blind spots for early-stage hepatocellular carcinoma during surveillance
Source: PLoS One. 2022 Sep 16;17(9):e0274747. doi: 10.1371/journal.pone.0274747 (PMC9481035; doi:10.1371/journal.pone.0274747)
Supplement: S1 File — (DOCX) [file pone.0274747.s001.docx]

**Title:** Impact of ultrasonographic blind spots for early-stage hepatocellular carcinoma during surveillance

**Running title:** Ability of current surveillance to detect early-stage HCC

Junghwan Lee,^1¶^ Su Bee Park, ^1¶^ Soyoung Byun,^1^ and Ha Il Kim^2^**^*^**

^1^Department of Internal Medicine, Asan Medical Center, Seoul, South Korea

^2^Division of Gastroenterology, Department of Internal Medicine, Kyung Hee University Hospital at Gangdong, Seoul, South Korea

***Corresponding author:** Ha Il Kim, M.D., Ph.D.

Division of Gastroenterology, Department of Internal Medicine, Kyung Hee University Hospital at Gangdong, Seoul, Korea, 892 Dongnam-ro, Gangdong-gu, 05278, Seoul, Republic of Korea, Telephone: +82-2-440-6220; Email: [mondosewan@gmail.com](mailto:mondosewan@gmail.com)

^¶^ Junghwan Lee and Su Bee Park contributed equally to this work.

**Table of Contents**

Supplementary Table 1 -------------------------------------------------------------------------------3

Supplementary Table 2 -------------------------------------------------------------------------------4

Supplementary Fig 1-----------------------------------------------------------------------------------5

Supplementary Table 3 -------------------------------------------------------------------------------6

**Supplementary Table 1.** Distribution of hepatocellular carcinoma <2cm by characteristic and blind spot location on ultrasonography

|  | **US-detected**  **group** | **US-missed**  **group** | ***P* value** |
| --- | --- | --- | --- |
| **All HCC tumors < 2cm** | n = 493 | n = 107 |  |
| **HCC <2cm within blind spots** | 182 (37.1%) | 64 (59.8%) | <0.001 |
| 1) hepatic dome | 63 (34.6%) | 22 (34.4%) |  |
| 2) caudate lobe or around IVC | 9 (4.9) | 10 (15.6%) |  |
| 3) Beneath ribs < 1 cm | 108 (59.3%) | 28 (43.8%) |  |
| 4) Left lateral segment, surface | 2 (1.1%) | 4 (6.3%) |  |

Abbreviations: HCC, hepatocellular carcinoma; IVC, inferior vena cava; US, ultrasonography

**Supplementary Table 2.** Tumor locations associated with the US-missed group

| **Tumor location *** | **Unadjusted** | | **Adjusted**† | |
| --- | --- | --- | --- | --- |
|  | **OR (95% CI)** | ***P* value** | **OR (95% CI)** | ***P* value** |
| Non-blind area on US | 1.0 (reference) |  | 1.0 (reference) |  |
| Blind spot 1 on US | 1.806 (1.226-2.660) | 0.003 | 1.903 (1.251-2.895) | 0.003 |
| Blind spot 2 on US | 6.481 (3.603-11.660) | <0.001 | 7.875 (4.029-15.391) | <0.001 |
| Blind spot 3 on US | 1.856 (1.277-2.699) | 0.001 | 1.643 (1.094-2.467) | 0.017 |
| Blind spot 4 on US | 4.188 (2.129-8.239) | <0.001 | 3.516 (1.655-7.471) | 0.001 |

* Definition of blind spots: hepatic dome (blind spot 1), caudate lobe or around the inferior vena cava (blind spot 2), <1 cm beneath the ribs (blind spot 3), and surface of the left lateral segment (blind spot 4).

†Adjusted for age ≥ 60 years, male sex, BMI ≥30 kg/m^2^, HBV infection, HCV infection, alcohol consumption, platelet count < 100k/mm^3^, serum AST > 40 IU/L, serum ALT >40 IU/L, serum AFP level >200ng/mL, surveillance performed at a tertiary referral hospital, HCC size ≥2 cm, cirrhosis on US, and fatty liver on US, which were variables meeting a prechosen *P* value (i.e., <0.1) in univariate analyses.

Abbreviations: OR, odds ratio; BMI, body mass index; HBV, hepatitis B infection; HCV, hepatitis C infection; AST, aspartate aminotransferase; ALT, alanine aminotransferase; AFP, alpha-fetoprotein; HCC, hepatocellular carcinoma; US, ultrasonography

**
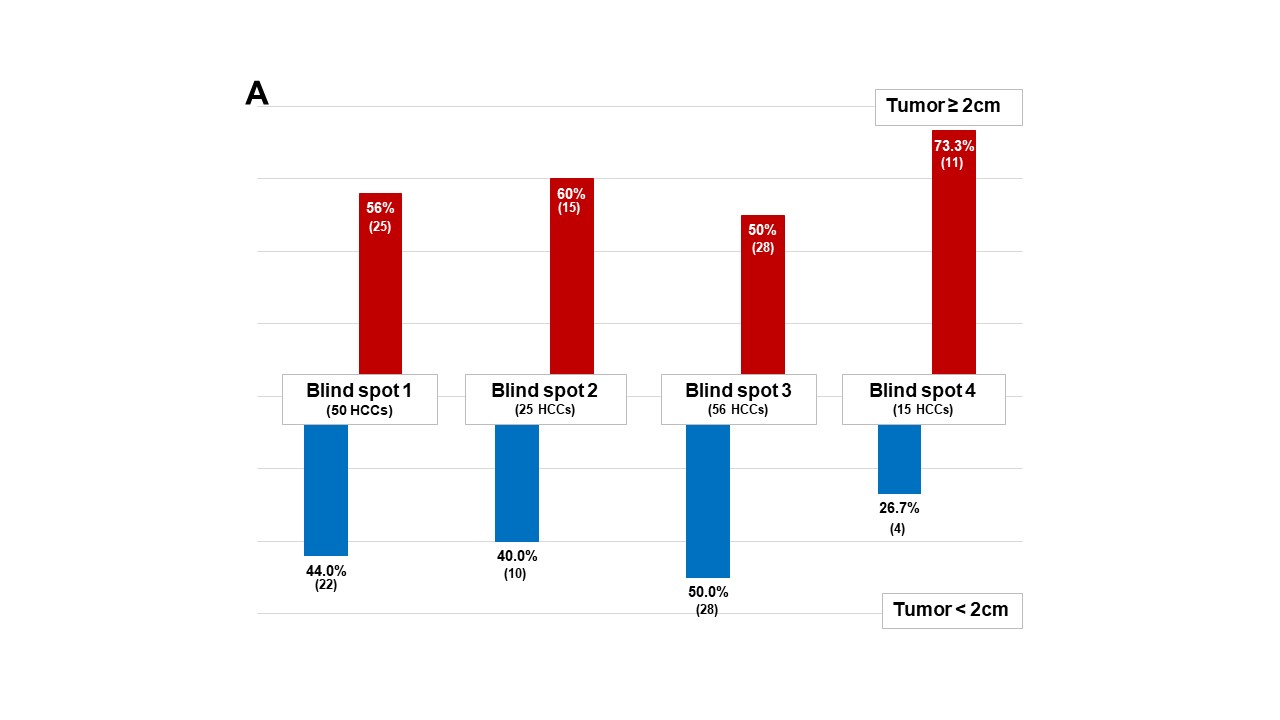

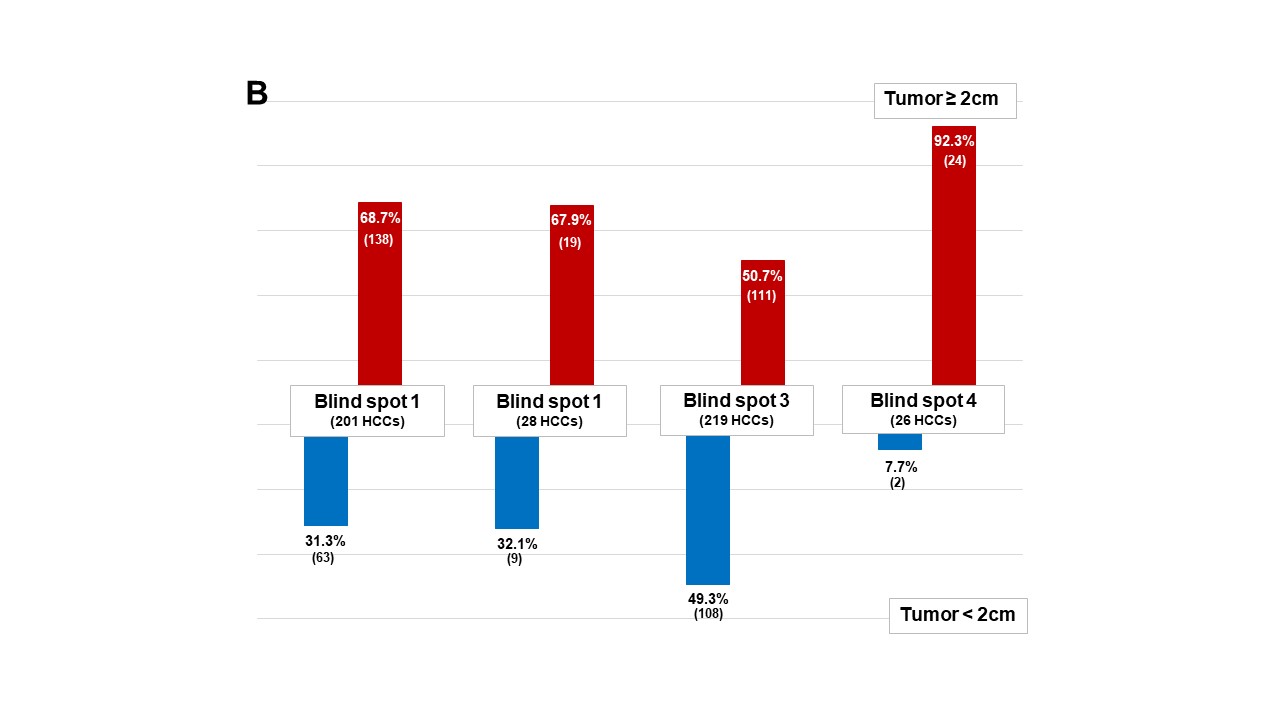
**

**Supplementary Fig 1.** Comparison of the size distribution of HCC in blind spots in the (A) US-missed and (B) US-detected group

HCC, hepatocellular carcinoma; US, ultrasonography. *Definition of blind spots: hepatic dome (blind spot 1), caudate lobe or around the inferior vena cava (blind spot 2), <1 cm beneath the ribs (blind spot 3), and surface of the left lateral segment (blind spot 4).

**Supplementary Table 3.** Factors associated with overall survival in patients with single-nodular HCC detected in blind spots

| **Variable** | **Unadjusted** | | **Adjusted** | |
| --- | --- | --- | --- | --- |
|  | **HR 95% CI** | ***P*** | **HR (95% CI)** | ***P*** |
| Age ≥ 60 years | 2.058 1.497-2.829 | <0.001 | 1.916 1.365-2.689 | <0.001 |
| Male sex | 1.033 0.728-1.465 | 0.856 |  |  |
| BMI ≥ 30 kg/m^2^ | 2.245 1.212-4.159 | 0.010 | 1.734 0.933-3.223 | 0.082 |
| HBV infection | 0.485 0.338-0.697 | <0.001 | 0.797 0.469-1.356 | 0.403 |
| HCV infection | 2.329 1.504-3.608 | <0.001 | 1.747 1.096-2.786 | 0.019 |
| Alcohol consumption | 1.033 0.744-1.434 | 0.846 |  |  |
| Platelet count < 100^3^/mm^3^ | 1.641 1.179-2.282 | 0.003 | 1.377 0.980-1.936 | 0.065 |
| Serum AST > 40 IU/L | 1.382 1.006-1.900 | 0.046 | 1.168 0.845-1.615 | 0.348 |
| Serum ALT > 40 IU/L | 1.167 0.847-1.607 | 0.344 |  |  |
| AFP >200 ng/mL | 1.396 0.964-2.022 | 0.077 | 1.186 0.792-1.777 | 0.408 |
| Surveillance at tertiary referral hospital | 1.005 0.730-1.384 | 0.973 |  |  |
| HCC tumor size ≥ 2 cm | 1.217 0.878-1.687 | 0.239 |  |  |
| Cirrhosis on US | 1.244 0.883-1.753 | 0.212 |  |  |
| Fatty liver on US | 0.982 0.577-1.674 | 0.948 |  |  |
| HCC missed by US | 1.571 1.123-2.198 | 0.008 | 1.413 1.003-1.990 | 0.048 |
| Initial curative treatment | 0.359 0.260-0.496 | <0.001 | 0.404 0.290-0.563 | <0.001 |

AFP, alpha-fetoprotein; ALT, alanine transaminase; AST, aspartate transaminase; BMI, body mass index; CI, confidence interval HBV, hepatitis B virus; HCC, hepatocellular carcinoma; HCV, hepatitis C virus; HR, hazard ratio; US, ultrasonography
